# soFusion: facilitating tissue structure identification via spatial multi-omics data fusion

**DOI:** 10.1093/bib/bbaf513

**Published:** 2025-09-29

**Authors:** Na Yu, Wenrui Li, Xue Sun, Jing Hu, Qi Zou, Zhiping Liu, Daoliang Zhang, Wei Zhang, Rui Gao

**Affiliations:** Center of Intelligent Medicine, School of Control Science and Engineering, Shandong University, No. 17923, Jingshi Road, Lixia District, Jinan, Shandong 250061, China; School of Life Sciences, Westlake University, No. 18, Shilongshan Road, Xihu District, Hangzhou, Zhejiang 310024, China; MOE Key Lab of Bioinformatics and Bioinformatics Division of BNRIST, Department of Automation, Tsinghua University, No 30, Shuangqing road, Haidian district, Beijing 100084, China; Center of Intelligent Medicine, School of Control Science and Engineering, Shandong University, No. 17923, Jingshi Road, Lixia District, Jinan, Shandong 250061, China; Department of Pathology, Qilu Hospital, Shandong University, No. 107, Wenhua Xilu, Lixia District, Jinan, ShanDong 250012, China; Michigan Center for Translational Pathology, University of Michigan, 1500 E. Medical Center Dr., Rogel Cancer Center, Ann Arbor, MI 48109-0940, United States; Center of Intelligent Medicine, School of Control Science and Engineering, Shandong University, No. 17923, Jingshi Road, Lixia District, Jinan, Shandong 250061, China; Center of Intelligent Medicine, School of Control Science and Engineering, Shandong University, No. 17923, Jingshi Road, Lixia District, Jinan, Shandong 250061, China; Center of Intelligent Medicine, School of Control Science and Engineering, Shandong University, No. 17923, Jingshi Road, Lixia District, Jinan, Shandong 250061, China; Institute of Science and Technology for Brain-Inspired Intelligence, MOE Key Laboratory of Computational Neuroscience and Brain-Inspired Intelligence, MOE Frontiers Center for Brain Science, Fudan University, No. 825, Zhangheng Road, Pudong New Area, Shanghai 200433, China; Center of Intelligent Medicine, School of Control Science and Engineering, Shandong University, No. 17923, Jingshi Road, Lixia District, Jinan, Shandong 250061, China; Center of Intelligent Medicine, School of Control Science and Engineering, Shandong University, No. 17923, Jingshi Road, Lixia District, Jinan, Shandong 250061, China

**Keywords:** spatial multi-omics, representation learning, feature fusion, spatial domain identification, soFusion

## Abstract

The rapid advancement of spatial multi-omics technologies has opened new avenues for dissecting tissue architecture with unprecedented resolution. However, inherent disparities across omics modalities, such as differences in biological hierarchy and resolution, pose significant challenges for integrative analysis. To address this, we present soFusion, a method for representation learning on spatial multi-omics data that enables automated identification of tissue compartmentalization. soFusion employs a graph convolutional network (GCN) to extract latent embeddings from spatial omics profiles. To simultaneously capture both cross-modality relationships and modality-specific features, we introduce a novel strategy for intra- and inter-omics feature learning. Moreover, modality-specific decoders are designed to preserve the unique information embedded in each omics type. We evaluated soFusion on multiple datasets including gene expression, protein expression, and epigenetic features. Across all benchmarks, soFusion consistently outperformed existing methods in delineating anatomical structures and identifying spatial domains with improved continuity and reduced noise. Collectively, soFusion offers an effective solution for spatial multi-omics integration, substantially enhancing the robustness of spatial domain identification.

## Introduction

Spatial omics technologies, such as spatial transcriptomics, spatial proteomics, and spatial metabolomics, enable the tracking of cellular spatial positions while quantifying molecular characteristics within the cell, which have significantly enhanced our ability to explore the relationship between tissue functions and cellular distribution [1–5]. Currently, spatial omics technologies are advancing toward multi-omics co-mapping, making it possible to perform synchronized analysis of various omics signal at the spatial level. For instance, techniques like SPOTS [6], SM-Omics [7], Stereo-CITE-seq [8], and spatial CITE-seq [9] can simultaneously measure spatial gene expression and surface protein expression in tissue sections, while spatial ATAC-RNA-seq and spatial CUT&Tag-RNA-seq can capture traits from both the transposase-accessible chromatin and transcriptome [10]. Moreover, the DBiT ARP-seq and DBiT CTRP-seq technologies take it a step further by enabling the spatial co-detection of the epigenome, transcriptome, and proteome in the same tissue section [11]. These breakthroughs in spatial multi-omics not only offer new insights into cell biology, but also provide unprecedented opportunities to explore the spatial landscape of cells within tissues.

In spatial omics research, integrating multi-omics data to accurately depict the spatial organization of complex tissues is crucial [1, 12]. Although spatial multi-omics data provide descriptions of cell states at various molecular levels, integrating these heterogeneous data still faces many challenges. First, the inherent differences among various omics make it extremely difficult to directly model the relationships between them. For example, genomics and epigenomics reflect the genetic blueprint of cells and the regulatory mechanisms of gene expression, while transcriptomics and proteomics represent the intermediate products and the final outcomes of gene expression, respectively. Although it is known that DNA is transcribed into RNA and RNA is subsequently translated into proteins, various factors that affect the flow of information (such as epigenetic modifications, changes in chromatin structure, and the actions of transcription factors) have not been fully elucidated. Therefore, accurately modeling the relationships among different omics layers in spatial omics data remains a significant problem. Second, the omics data themselves differ in resolution and background distribution. For example, differences in feature counts (e.g. the number of measured proteins versus transcripts) and statistical distributions among omics make the fusion of different features complex [13]. Moreover, uniformly encoding the features of different omics and their corresponding spatial information to create a cohesive low-dimensional representation is key for precise domain identification [14–16].

In recent years, methods for integrating multi-omics data have been proposed. Currently, most approaches focus on integrating single-cell multi-omics data, such as Seurat WNN [17], MultiVI [18], totalVI [19], MOFA+ [20], scMM [21], and StabMap [22]. However, these methods do not fully utilize the location information in spatial multi-omics, resulting in low accuracy for spatial domain identification. To address this issue, several methods based on spatial multi-omics data have been proposed in recent years. SpatialGlue [23], the first spatial multi-omics integration method, employs a graph neural network with a dual attention mechanism to integrate different omics data and decode spatial domains. However, this method mainly focuses on the complementary information among omics and does not fully consider tailored modeling for each omics. soScope [24] is a unified generative model that simulates the generation process of different spatial omics data. Nevertheless, it requires morphological images of the same tissue as input, which may affect computational efficiency in large-scale analyses. PRESENT [25] is an efficient and scalable contrastive learning framework that captures spatial dependencies and the complementarity of multi-omics information through low-dimensional representations. In addition, SSGATE [26] is an integration method based on a dual-path graph attention autoencoder i.e. specifically designed for integrating spatial transcriptomics and spatial proteomics. spaMultiVAE [27] uses a dependency-aware generative variational autoencoder framework for cross-modal representation and is also applied to the integration of transcriptomics and proteomics. Although these methods have shown potential in integrating spatial multi-omics data, they do not comprehensively account for the distribution characteristics of different omics. As a result, the latent representations do not fully exploit the intrinsic associations and specificities among the different omics, which may affect the robustness and accuracy for spatial domain identification.

To full fill these gaps, we propose a method named soFusion that can efficiently integrate multiple types of spatial omics data. First, soFusion extracts embeddings from two sets of spatial omics data using a graph convolutional network (GCN). To generate a joint representation that both preserves the associations between different omics and captures their individual specificities, we introduce a novel intra- and inter-omics feature learning strategy. Additionally, to better reflect the distribution patterns of different omics data, we model the count features of transcriptomic, epigenomic, and proteomic data using zero-inflated negative binomial (ZINB) distribution, Bernoulli distribution, and negative binomial mixture distribution, respectively. For other omics data, we design a general decoder based on a fully connected network. Benefiting from the innovative intra- and inter-omics feature learning strategy and the adaptive decoder based on data characteristics, soFusion can capture the relationships among different omics features and generate an effective representation for spatial multi-omics data.

To evaluate the capability of soFusion in representing spatial multi-omics data, we tested it on six datasets generated by different technologies. Compared with other methods, soFusion exhibited higher accuracy and lower noise in spatial domain identification. In datasets from the mouse thymus and brain, soFusion captured more detailed anatomical structures. In the mouse spleen dataset, soFusion successfully identified regions enriched with different immune cell types. In the human tonsil dataset, soFusion accurately distinguished the light and dark zones of the germinal center (GC). Overall, soFusion demonstrated outstanding performance in integrating spatial multi-omics data, providing a new technical pathway for analyzing tissue structure and function.

## Materials and methods

### Overview of soFusion

**Figure 1:**
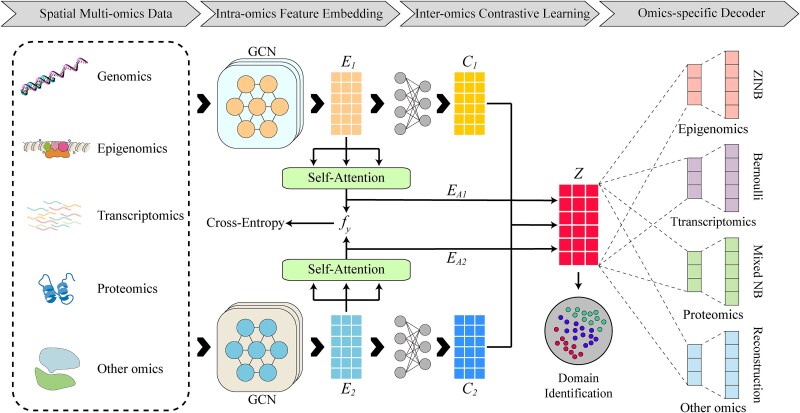
The overall workflow of soFusion. Firstly, the GCN is used for intra-omics feature embedding. Then, the multi-omics features are integrated, and the inter-omics contrastive learning is used to capture the commonality between the omics. Finally, different decoders are utilized to reconstruct the features of each omics. The joint representation learned by soFusion can be further applied to spatial domain recognition.

soFusion consists of three main components. The first component is a GCN module for the embedding intra-omics features. The second component is an inter-omics feature integration module that employs a contrastive learning strategy. The third component is a decoder module for different omics features. The learned multi-omics representations $Z$ is used for spatial domain identification. The overall workflow of soFusion is shown in Fig. 1.

### Multi-omics data input for soFusion

soFusion enables the integration of any two modalities within spatial multi-omics datasets. soFusion accepts three inputs: two feature matrices (denoted as ${X}_1\in{R}^{N\times{d}_1}$ and ${X}_2\in{R}^{N\times{d}_2}$) representing multi-omics measurements, along with a spatial coordinate matrix $M\in{R}^{N\times 2}$ containing physical locations of $N$ spots or cells. ${d}_1$ and ${d}_2$ indicate the feature dimensions of respective omics modalities. Then, the feature matrices undergo standardized preprocessing, while an undirected weighted graph is constructed based on the coordinate matrix $M$. For any pair of spots or cells, the closer their spatial distance, the greater their similarity. Therefore, the adjacency matrix between any two spots is defined as follows:


(1) \begin{equation*} {A}_{ij}=\mathit{\exp}\left(-\frac{d{\left(i,j\right)}^2}{2{\lambda}^2}\right), \end{equation*}

where $d\left(i,j\right)$ is the Euclidean distance between spots $i$ and $j$. $\lambda$ is a hyperparameter that controls the relationship between the edge weight and the distance.

### Feature embedding for the intra-omics

We first adopt GCNs [28] in soFusion to encode features from each omics, obtaining the low-dimensional embeddings respectively. GCN can capture cellular expression patterns and the characteristics of neighboring microenvironments [23]. By iteratively passing and aggregating the information from neighboring spots, GCN learns the dependencies between spots or cells. Specifically, for features of each omics, this process is denoted as follows:


(2) \begin{equation*} {\displaystyle \begin{array}{c}{E}^{\left(l+1\right)}={\overset{\sim }{D}}^{-\frac{1}{2}}\overset{\sim }{A}{\overset{\sim }{D}}^{-\frac{1}{2}}{E}^{(l)}{W}^{(l)},\end{array}} \end{equation*}

where ${E}^{(l)}$is the input feature matrix of the $l$-th GCN layer, with the initial input ${E}^{(0)}$ corresponding to the expression matrices ${X}_1$ or ${X}_2$. $\overset{\sim }{A}=A+I$, and $I$ is the identity matrix. $\overset{\sim }{D}$ and ${W}^{(l)}$ represent the weight matrix and learnable parameters. The embeddings of two omics obtained through GCN encoder are denoted as ${E}_1$ and ${E}_2$, respectively.

To further enhance the representation ability of the embeddings, soFusion adopts the attention mechanism for learning the global relationship between spots or cells:


(3) \begin{equation*} {\displaystyle \begin{array}{c}{E}_A= softmax\left(\frac{E\bullet{E}^T}{\sqrt{g}}\right)\bullet E\end{array}} \end{equation*}

where $g$ is the dimension of the embedding space, and $E$ represents the embedding ${E}_1$ or ${E}_2$ obtained by GCN. ${E}_A$ corresponds to the new features ${E}_{A1}$ or ${E}_{A2}$ for each omics.

Then, to preserve the unique biological characteristics within each modality as much as possible, we employed a novel label prediction strategy. In brief, the embedding features ${E}_{A1}$ and ${E}_{A2}$ from different omics are concatenated to form a feature set ${E}_{All}$. A fully connected network is then used to predict the omics labels of ${E}_{All}$, and the difference between the predicted and true labels is quantified using a Cross-Entropy loss, which guides model training:


(4) \begin{equation*} {\displaystyle \begin{array}{c}{L}_{CE}= CE\left({f}_y\left({E}_{All}\right),Y\right).\end{array}} \end{equation*}

$CE\left(\bullet \right)$ represents the cross-entropy. ${f}_y\left(\bullet \right)$ is the fully connected network. $Y$ is the labels for omics and constructed based on the feature source.

### Contrastive learning for inter-omics features integration

Single-omics data can only describe tissue characteristics from a specific perspective, while multi-omics data allow for a more comprehensive understanding of the complexity of tissue from multiple dimensions and levels, such as the interactions between genes, proteins, and metabolites. However, inherent differences between omics and variations in data resolution and background distribution significantly hinder the effective integration and analysis of multi-omics data.

Considering that different omics contain both unique information and exhibit similarities as well as correlations, soFusion employs a cross-omics contrastive learning strategy to enhance the fusion of commonality between omics. First, soFusion uses a fully connected network to map the embedding features ${E}_1$ and ${E}_2$ into a unified space:


(5) \begin{equation*} {\displaystyle \begin{array}{c}C= Rule\left({W}_CE+{b}_C\right).\end{array}} \end{equation*}

${W}_C$ and ${b}_C$ are the parameters of the fully connected network. $E$ represents the embeddings of two omics, ${E}_1$ or ${E}_2$. $C$ corresponds to ${C}_1$ or ${C}_2$. ${C}_1$ and ${C}_2$ are further integrated using a fully connected network:


(6) \begin{equation*} {\displaystyle \begin{array}{c}{E}_C={W}_E\bullet concat\left({C}_1,{C}_2\right)+{b}_E,\end{array}} \end{equation*}

where ${W}_E$ and ${b}_E$ are the parameters of the fully connected network.

In this process, soFusion uses the following constraint to enhance the commonality of the two omics:


(7) \begin{equation*} {\displaystyle \begin{array}{c}{L}_{con}={\left\Vert{\overset{\sim }{C}}_1{\overset{\sim }{C}}_1^T-{\overset{\sim }{C}}_2{\overset{\sim }{C}}_2^T\right\Vert}_2^2\end{array}} \end{equation*}

where ${\overset{\sim }{C}}_1$ and ${\overset{\sim }{C}}_2$ are the normalized forms of ${C}_1$ and ${C}_2$, respectively.

Then, soFusion obtains the multi-omics representation in the following way:


(8) \begin{equation*} {\displaystyle \begin{array}{c}Z=\alpha{E}_{A1}+\beta{E}_{A2}+\gamma{E}_C.\end{array}} \end{equation*}

${E}_{A1}$ and ${E}_{A2}$ are the embedding features of the two spatial omics obtained from Equation (3), and ${E}_C$ represents the cross-omics feature obtained through Equation (6). $\alpha$, $\beta$ and $\gamma$ are hyperparameters that control the importance of different features.

To further ensuring that spots or cells with close spatial distances are close to each other in the embedding space, we construct the following constraint loss:


(9) \begin{equation*} {\displaystyle \begin{array}{c}{L}_{reg}=\frac{\sum_{i=1}^N\sum_{j=1}^N{D}_{ij}\left(1-{Q}_{ij}\right)}{N^2}.\end{array}} \end{equation*}

$D$ is obtained by calculating the Euclidean distance between the spatial coordinates of the spots or cells. $Q$ is calculated using the Euclidean distance between the multi-omics representation of the spots.

### Omics-specific decoders

Finally, soFusion reconstructs the original features for each omics from the multi-omics representation. Due to the differing background distributions of the data for each omics, the decoders need to be omics-specifically customized. For example, protein data from antibody-derived tags (ADTs) typically have low dimensionality and low sparsity, while transcriptomic data exhibit high dimensionality, high sparsity, and a higher dropout rate. In contrast, epigenomic data obtained through ATAC-seq shows higher dimensionality, sparsity, and dropout rates [29–31].

The ZINB distribution is an effective model for describing the distribution characteristics of gene expression data [30, 32]. soFusion uses ZINB to fit the expression matrix ${X}^{RNA}\in{R}^{N\times{d}_1}$. First, the parameters of the ZINB distribution are estimated using fully connected networks:


(10) \begin{equation*} {\displaystyle \begin{array}{c}{\pi}_i= sigmoid\left({W}_{\pi }{f}_D^{ZINB}\left({Z}_i\right)\right),\end{array}} \end{equation*}

(11) \begin{equation*} {\displaystyle \begin{array}{c}{\mu}_i=\mathit{\exp}\left({W}_{\mu }{f}_D^{ZINB}\left({Z}_i\right)\right),\end{array}} \end{equation*}

(12) \begin{equation*} {\displaystyle \begin{array}{c}{\theta}_i=\mathit{\exp}\left({W}_{\theta }{f}_D^{ZINB}\left({Z}_i\right)\right).\end{array}} \end{equation*}

$\pi$ , $\mu$, and $\theta$ represent the dropout probability, mean, and dispersion of the ZINB distribution, respectively. ${f}_D^{ZINB}\left(\bullet \right)$ is the decoder. ${W}_{\pi }$, ${W}_{\mu }$, and ${W}_{\theta }$ represent the learnable weight matrix.

Then, the negative log-likelihood of the ZINB distribution is used as the reconstruction loss for the expression component:


(13) \begin{equation*} {L}_{RNA}=-\mathit{\log}\sum_{i=1}^N\left( ZINB\left({X}_i^{RNA}|{\pi}_i,{\mu}_i,{\theta}_i\right)\right). \end{equation*}

ATAC-seq data is used to depict chromatin accessibility, characterized by highly sparse binarized observations that reflect the open state of genome regions [33]. soFusion models the ATAC-seq data ${X}^{ATAC}\in{R}^{N\times{d}_2}$ using Bernoulli distribution [27]:


(14) \begin{equation*} {\displaystyle \begin{array}{c}{X}_{iq}^{ATAC}\sim Bernoulli\left({p}_{iq}\right).\end{array}} \end{equation*}

$i$ is the index of the spot. $q$ is the index of ATAC-seq data. $p$ is the parameter of the Bernoulli distribution, estimated by the decoder:


(15) \begin{equation*} {\displaystyle \begin{array}{c}{p}_i^{ATAC}= sigmoid\left({f}_p\left({f}_D^{ATAC}\left({Z}_i\right)\right)\right),\end{array}} \end{equation*}

where ${f}_p\left(\bullet \right)$and ${f}_D^{ATAC}\left(\bullet \right)$ are decoder networks.

Finally, the reconstruction loss for the ATAC component is defined as:


(16) \begin{equation*} {L}_{ATAC}=\sum_{i=1}^N BCE\left({p}_i,{X}_i^{ATAC}\right), \end{equation*}

where $BCE\left(\bullet \right)$ is the Binary Cross-Entropy.

The protein measurement process involves barcode-tagged antibodies to bind to specific proteins, but the binding of antibodies and antigens is not always specific. Nonspecific binding can lead to background signals, making its analysis more complicated. Similar to SpaMultiVAE and totalVI, soFusion models protein counts ${X}^{ADT}\in{R}^{N\times{d}_3}$ by mixing two negative binomial distributions:


(17) \begin{align*} p\left({X}^{ADT}|Z\right)=&\prod_i MixtureNB\left({X}_i^{ADT}|{\nu}_i^b,{\nu}_i^f,{\varphi}^{ADT},{\pi}_i^{ADT}\right)\nonumber\\=&\prod_i{\pi}_i\times NB\left({X}_i^{ADT}|{\nu}_i^b,{\varphi}^{ADT}\right)+\left(1-{\pi}_i\right)\nonumber\\&\times NB\left({X}_i^{ADT}|{\nu}_i^f,{\varphi}^{ADT}\right). \end{align*}

In this equation, ${\nu}_i^b$ is the background component of the protein counts. ${\nu}_i^f$ is the foreground component. ${\pi}_i^{ADT}$ denotes the background probability, and ${\varphi}^{ADT}$ refers to the dispersion. ${\varphi}_p^{ADT}$ is a trainable parameter of the protein $p$.

For ${\nu}_{ip}^b$，we assume it follows a prior log-normal distribution:


(18) \begin{equation*} {\displaystyle \begin{array}{c}p\left({\nu}_{ip}^b\right)= lognormal\left({m}_p^{prior},{\sigma}_p^{prior}\right),\end{array}} \end{equation*}

where $i$ and $p$ represent the indices of the spots and proteins, respectively. ${m}_p^{prior}$ and ${\sigma}_p^{prior}$ are the learnable prior parameters for the background intensity. To obtain these two parameters, a two-component Gaussian mixture model (GMM) is first fitted to the log-transformed counts of each protein $p$, and the smaller component of the GMM is used as the initial value.

The posterior distribution of ${\nu}_i^b$ is inferred by the neural network.


(19) \begin{equation*} {\displaystyle \begin{array}{c}{m}_i^b={f}_{m^b}\left({f}_D^{ADT}\left({Z}_i\right)\right),\end{array}} \end{equation*}

(20) \begin{equation*} {\displaystyle \begin{array}{c}{\sigma}_i^b=\mathit{\exp}\left({f}_{\sigma^b}\left({f}_D^{ADT}\left({Z}_i\right)\right)\right),\end{array}} \end{equation*}

(21) \begin{equation*} {\displaystyle \begin{array}{c}q\left({\nu}_i^b|{Z}_i\right)= lognormal\left({m}_i^b,{\sigma}_i^b\right).\end{array}} \end{equation*}

${\pi}_i^{ADT}$ controls the probability of the background intensity, also estimated by the decoder network:


(22) \begin{equation*} {\displaystyle \begin{array}{c}{\pi}_i^{ADT}= sigmoid\left({f}_{\pi^{ADT}}\left({f}_D^{ADT}\left({Z}_i\right)\right)\right).\end{array}} \end{equation*}

To ensure that the foreground is greater than the background, the following operation is performed:


(23) \begin{equation*} {\displaystyle \begin{array}{c}{\nu}_i^f=\left(1+{\alpha}_i^{ADT}\right)\times{\nu}_i^b,\end{array}} \end{equation*}

where ${\alpha}_i^{ADT}$is inferred by the neural network:


(24) \begin{equation*} {\displaystyle \begin{array}{c}{\alpha}_i^{ADT}= softplus\left({f}_{\alpha^{ADT}}\left({f}_D^{ADT}\left({Z}_i\right)\right)\right).\end{array}} \end{equation*}

In Equations (19), (20), (22), and (24), ${f}_{m^b}\left(\bullet \right)$, ${f}_{\sigma^b}\left(\bullet \right)$, ${f}_{\pi^{ADT}}\left(\bullet \right)$and ${f}_{\alpha^{ADT}}\left(\bullet \right)$ represent different neural networks, ${f}_D^{ADT}\left(\bullet \right)$is the decoder for the proteins.

Ultimately, the negative log-likelihood of the negative binomial mixture distribution is used as the reconstruction loss for the protein component:


(25) \begin{equation*} {L}_{ADT}=-\mathit{\log}\sum_{i=1}^N MixtureNB\left({X}_i^{ADT}|{\nu}_i^b,{\nu}_i^f,{\varphi}^{ADT},{\pi}_i^{ADT}\right). \end{equation*}

In addition to RNA, ATAC, and ADT, soFusion can also be applied to the analysis of other omics, such as metabolomics. To this end, soFusion constructs a universal decoder based on the fully connected network for the reconstruction of other omics data ${X}^O\in{R}^{N\times{d}_4}$, and uses the cross-entropy loss as the reconstruction loss:


(26) \begin{equation*} {L}_O=\sum_{i=1}^N CE\left({f}_D^O\left({Z}_i\right),{X}_i^O\right). \end{equation*}

where ${f}_D^O\left(\bullet \right)$ denotes the fully connected network, and $CE\left(\bullet \right)$ represents the Cross-Entropy.

Therefore, the reconstruction loss of soFusion can be denoted as:


(27) \begin{equation*} {\displaystyle \begin{array}{c}{L}_{Recon}={L}_{RNA}+{L}_{ATAC}+{L}_{ADT}+{L}_o.\end{array}} \end{equation*}

### Overall loss functions of soFusion

In summary, the loss function of soFusion consists of four parts, as shown in Equations (4), (7), (9), and (27), and can be expressed as:


(28) \begin{equation*} {\displaystyle \begin{array}{c}L=a{L}_{CE}+b{L}_{con}+c{L}_{reg}+d{L}_{Recon}.\end{array}} \end{equation*}

where $a$, $b$, $c$, and $d$ are hyperparameters that control the weights of different losses, with default values set to 1.

### Spatial clustering

Based on the multi-omics representations $Z$ learned by soFusion, we applied both the K-means and Louvain methods for clustering. For datasets where the number of spatial domains is known, we used the K-means algorithm, with the number of clusters set to match the ground truth. For datasets where the number of spatial domains is unknown, soFusion uses the Louvain algorithm for spatial clustering. Additionally, we can incorporate prior biological knowledge—such as known anatomical structures, cell types, or marker gene expression patterns—to guide the optimal number of spatial domains.

### Datasets and benchmark methods

We validated soFusion using six datasets obtained from different technologies: Stereo-CITE-seq data from mouse thymus [8], SPOTS data from mouse spleen [6], Spatial-CITE-seq data from human tonsil [9], 10× Visium RNA-protein data from human lymph node [23], MISAR-seq data from mouse brain [34], as well as Spatial ATAC-RNA-seq and spatial CUT&Tag-RNA-seq data from mouse (P22) brain [10]. All datasets are publicly available, and the detailed information for each dataset can be found in the Supplementary Materials.

We compared soFusion with five advanced multi-omics methods, including two integration methods for nonspatial multi-omics, MultiVI [18], and Seurat WNN [17], as well as three methods designed for spatial multi-omics, SpatialGlue [23], PRESENT [25], and spaMultiVAE [27].

## Results

### soFusion enables precise delineation of the intricate architecture within murine thymic microenvironments

The spatial heterogeneity of thymic tissue makes its structural analysis particularly complex [35]. However, spatial multi-omics technologies provide unprecedented resolution for studying intricate organ structures. Spatial transcriptomics of the thymus helps not only to reveal the relationship between the thymic microenvironment and immune function, but also offers new approaches for investigating immune-related diseases. We applied multiple methods to decipher the complex structure of a mouse thymus dataset obtained using the Stereo-CITE-seq technology [8].

**Figure 2:**
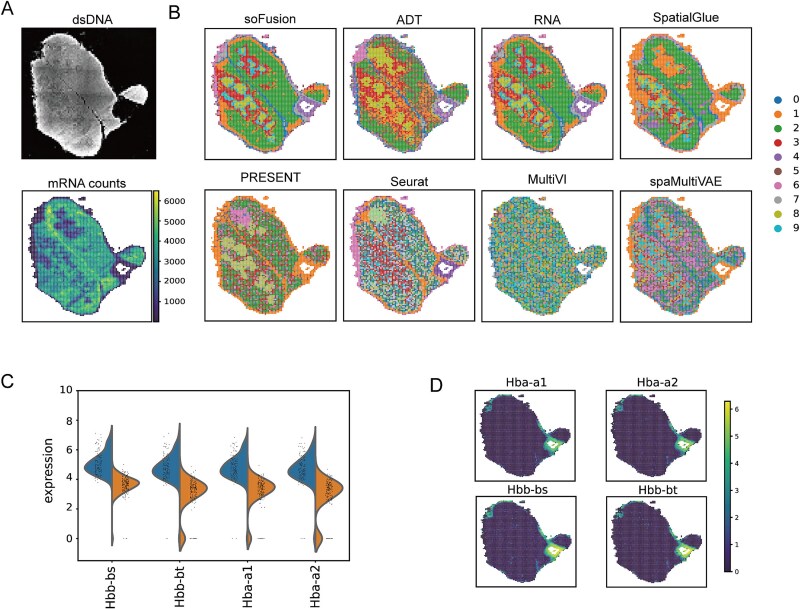
Performance comparisons of different methods on the mouse thymus dataset acquired with Stereo-CITE-seq. (A) dsDNA image [23] and total mRNA counts. (B) The clustering results of soFusion, SpatialGlue, PRESENT, Seurat, MultiVI, and spaMultiVAE methods, as well as the results of soFusion using only ADT or RNA. (C) Violin plot showing the top 4 differentially expressed genes between domains 4 and 7. (D) Expression of the 4 differentially expressed genes.

Stereo-CITE-seq technology captures both mRNA and protein information of mouse thymus tissue at subcellular resolution. Fig. 2A shows the images of dsDNA and total mRNA counts. In Fig. 2B, we present the spatial domain identification results from six methods. It is evident that the clustering results of soFusion are highly consistent with the dsDNA and total mRNA count images (Fig. 2A and B). For instance, soFusion accurately identified the medulla (Cluster 8), inner cortex (Cluster 9), middle cortex (Cluster 3), outer cortex (Cluster 2), and subcapsular region (Cluster 1) of the thymus. Compared to PRESENT, Seurat, MultiVI, and spaMultiVAE, soFusion demonstrates a more compact and continuous spatial domain identification across the entire slice. While PRESENT and spaMultiVAE could separate the medulla and cortex, significant noise was still present in many regions, especially in the outer cortex. MultiVI and Seurat showed poorer identification results. Moreover, soFusion and SpatialGlue provided clearer and more accurate delineations of the medulla and inner, middle, and outer cortices. soFusion also demonstrated higher precision than SpatialGlue in the structural delineation of the right upper lobe. Additionally, we show the spatial domain identification results of soFusion using only RNA and ADT data (Fig. 2B). The results indicate that compared to protein data, RNA data identified spatial domains with lower noise and provided more accurate thymus structural delineation. This suggests that significant gene expression differences exist between the thymic medulla and cortex regions, and the high resolution of RNA data aids in more precise thymus structure identification.

Next, we conducted further analysis of the results obtained from soFusion. According to soFusion, both domain 4 and domain 7 belong to connective tissue, but soFusion classified them as two distinct regions. We analyzed and visualized the differentially expressed genes between these two regions and found that the expression levels of *Hba-a1*, *Hba-a2*, *Hbb-bs*, and *Hbb-bt* were significantly higher in domain 4 (Fig. 2C and D). The HBA and HBB genes encode the α and β chains of human hemoglobin, respectively, and play key roles in hemoglobin synthesis and oxygen transport [36]. Therefore, domain 4 is likely to be rich in red blood cells and closely related to the thymus’s blood supply or oxygen regulation. However, this phenomenon is not clearly observed in Fig. 2A. Therefore, by integrating multi-omics data, soFusion can precisely identify tissue domains that are not revealed by individual omics data. Through high-resolution spatial transcriptomics analysis, soFusion not only improves the ability to detect subtle tissue differences but also provides new insights into the complex functional region delineation of organs.

### soFusion isolates regions of the mouse spleen enriched with different immune cells

**Figure 3:**
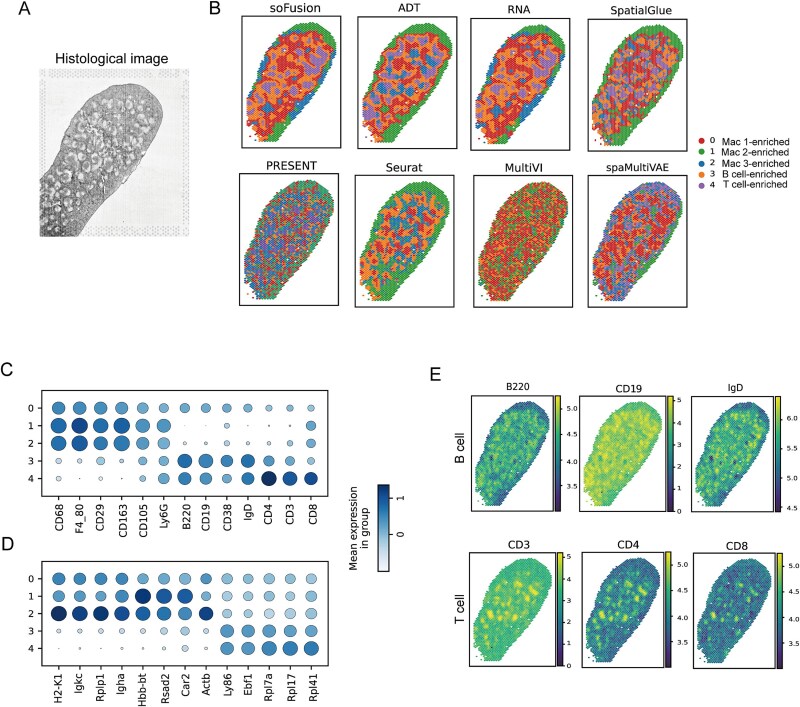
Performance comparisons of different methods on the SPOTS mouse spleen dataset. (A) The histological image of mouse spleen. (B) The clustering results of soFusion, SpatialGlue, PRESENT, Seurat, MultiVI, and spaMultiVAE methods, as well as the results of soFusion using only ADT or RNA. (C) Expression pattern of marker ADTs across different domains. (D) Expression pattern of marker genes across different domains. (E) Expression levels of B and T cell markers.

The spleen, as the largest secondary lymphoid organ in the human body (Fig. 3A), plays a crucial role in immune response regulation and blood filtration [37]. Its complex cellular composition and intricate spatial structure determine the interactions and functional localization of immune cells. Therefore, studying the spatial distribution of different immune cells in the spleen helps reveal the functional partitioning of these cells within the organ. In this study, we utilized a mouse spleen multi-omics dataset, generated by SPOTS technology (integrating spatial proteomics and transcriptomics data), to systematically analyze the spatial distribution patterns of immune cells.

First, we compared the spatial domain results identified by different methods (Fig. 3B). soFusion successfully identified five distinct immune cell-enriched regions: a T cell-dominant region (domain 4), a B cell-enriched region (domain 3), and three macrophage subpopulation regions (domains 0–2). In comparison, SpatialGlue and Seurat shows clear spatial localization disruptions. PRESENT, MultiVI, and spaMultiVAE exhibits higher clustering dispersion. Notably, using only ADT and RNA data, we observed similar immune cell distributions, such as the identification of the Mac-enriched region (Fig. 3B). We also conducted supplementary experiments on another SPOTS-based dataset of mouse spleen, where soFusion again produced more continuous clustering results than other methods (Supplementary Fig. S1).

To further verify the main cell types in each region, we performed differential expression analysis on the transcriptomic and ADT data (Fig. 3C and D). The results showed that T cell markers (CD3, CD4, and CD8) were highly expressed in domain 4, while B cell markers (B220, CD19, and IgD) were significantly enriched in domain 3 (Fig. 3E) [6, 38]. Thus, these two regions were identified as T cell- and B cell-enriched regions, respectively. Macrophage markers (such as CD68, CD163, and F4_80) were highly expressed across all three macrophage subpopulations [6]. Notably, although Mac2 and Mac3 displayed similar ADT expression profiles, soFusion successfully differentiated these subpopulations using transcriptomic features. For example, Mac2 specifically expressed the *Hbb-bt*/*Rsad2*/*Car2* gene cluster, suggesting it may be located in the red pulp and involved in erythrocyte metabolism [37, 39]. In contrast, Mac3 was enriched in antigen-presentation-related genes, such as *H2-K1* and *Rplp1*, along with co-expression of immunoglobulin genes like *Igkc* and *Igha*.

Therefore, the unique cross-omics information integration mechanism in soFusion not only enables precise immune microenvironment analysis but also provides a new technical paradigm for spatial omics research in complex tissues.

### soFusion accurately integrates multi-modal data from the human tonsil and lymph node

The tonsil is a critical component of the human immune system and plays a vital role in defending against foreign pathogens as well as maintaining immune homeostasis. Within the tonsil, the GC can be divided into the light and dark zones [40], which are key areas for B cell clonal expansion and antibody affinity maturation. We further applied spatial-CITE-seq technology to obtain spatial multi-omics data for spatial analysis of the human tonsil [9].

**Figure 4:**
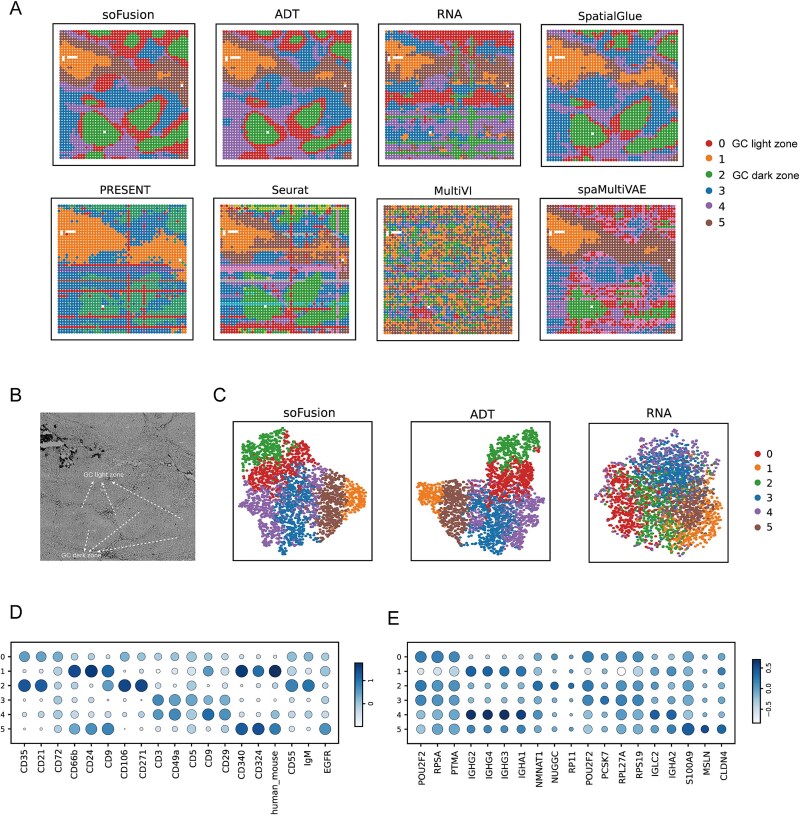
Performance comparisons of different methods on the human tonsil data acquired with spatial-CITE-seq. (A) The clustering results of soFusion, SpatialGlue, PRESENT, Seurat, MultiVI, and spaMultiVAE methods, as well as the results of soFusion using only ADT or RNA. (B) The histological image of human tonsil [27]. (C) The UMAP visualization of the latent representations. (D) The dot plot demonstrating the differences in ADT expression levels. (E) The dot plot demonstrating the difference in RNA expression levels.

Similar to studies conducted above, we compared the results of different methods and analyzed the effects of using separate ADT or RNA data in soFusion (Fig. 4A). As shown in Fig. 4A, soFusion again achieved the smoothest and continuous clustering results on this dataset, which were highly consistent with the tissue image (Fig. 4B). Among the six methods, MultiVI produced the worst clustering results, failing to identify the spatial structure of the tonsil. PRESENT and Seurat performed better than MultiVI but still failed to distinguish the light zone of the GC, with stripe-like noise appearing within the clusters. In contrast, soFusion, SpatialGlue, and spaMultiVAE successfully separated the light and dark zone in GC. Fig. 4C shows the UMAP visualization of the embedding features for soFusion and single-omics data (ADT and RNA). We found that whether through spatial clustering or dimensionality reduction, ADT data were more useful for delineating the spatial domains of the tonsil, while RNA data performed poorly. This highlights the variation in the performance of different omics data in spatial domain recognition across tissues.

To further assess the differences in RNA and ADT expression levels between different domains, we conducted differential expression analysis again (Fig. 4D and E). We found that, compared to the GC light zone, the dark zone showed upregulated expression of several ADT markers such as CD35, CD21, CD106, CD271, and IgM. However, at the transcriptomic level, the differences between the two regions were minimal. Some studies suggest that despite small transcriptional differences, these subtle variations between the GC light and dark zones may still lead to significant functional differences, or that the functional differences between the two regions primarily rely on post-transcriptional mechanisms [41]. This finding further emphasizes the importance of multi-omics integration in revealing cellular functional characteristics.

We also applied soFusion to analyze the lymph node which is another important immune organ in the human body. This dataset was generated using 10× Genomics Visium RNA and protein co-profiling technology (Section A1) [23] and provided labels manually annotated by pathologists (Supplementary Fig. S2A). soFusion successfully identified two important structures (the cortex and capsule) and achieved the highest ARI value among all methods (Supplementary Fig. S2B). In contrast, the two nonspatial multi-omics integration methods, MultiVI and Seurat, produced poorer clustering results, highlighting the necessity of incorporating spatial information. We observed that the ARI values for all methods are relatively low, which may be attributed to the complexity and fine structure of the tissue in the lymph node dataset. Furthermore, compared to the results using only ADT or RNA, soFusion demonstrated superior clustering performance, proving its capability in spatial multi-omics information integration.

### soFusion accurately identifies the complex structures in mouse brain

**Figure 5:**
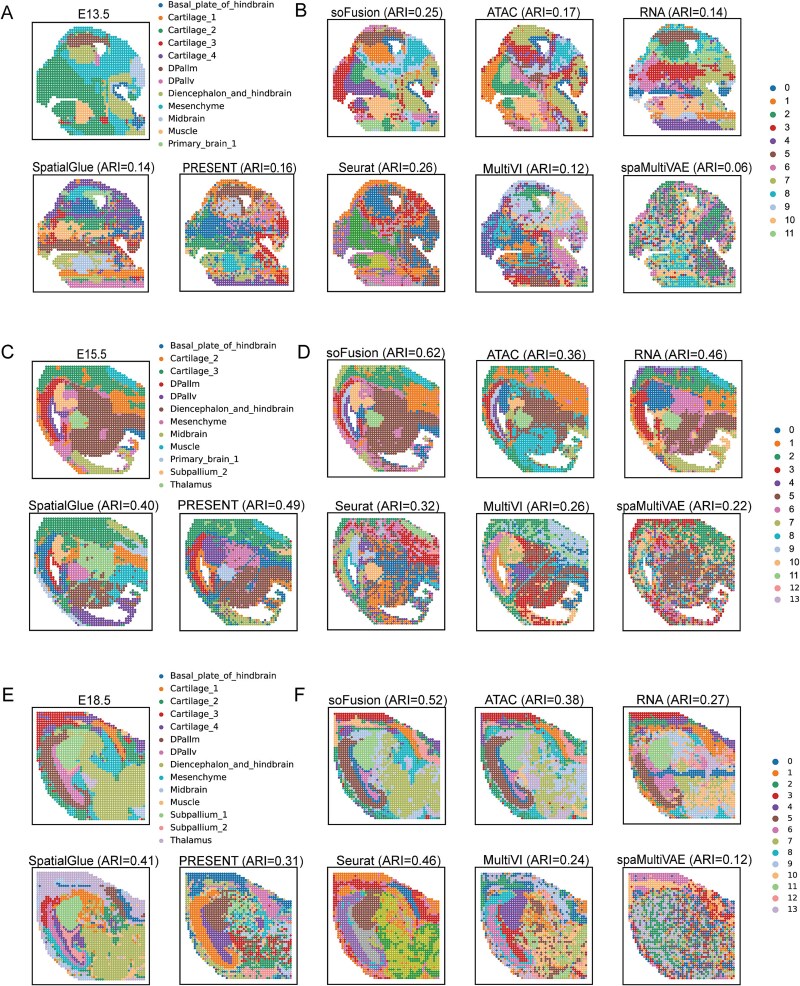
Performance comparisons of different methods on the MISAR-seq mouse brain dataset. (A) The manual annotation of the E13.5 sample. (B) Clustering results on E13.5 sample by different methods. (C) The manual annotation of the E15.5 sample. (D) Clustering results on E15.5 sample by different methods. (E) The manual annotation of the E18.5 sample. (F) Clustering results on E18.5 sample by different methods.

To further validate the capability of soFusion in integrating spatial multi-omics information, we adopted a time series dataset for mouse brain development using MISAR-seq [34]. This dataset not only includes paired data of spatial transcriptomics (RNA) and epigenomics (ATAC) but also captures the spatiotemporal dynamics across multiple critical developmental stages (E13.5, E15.5, E18.5, Fig. 5A, C, and  E).

Figure 5B, D and F illustrate the domain identification results of the different methods on this dataset. In the E13.5 sample (Fig. 5B), both soFusion and Seurat exhibited higher ARI values, with soFusion demonstrating greater accuracy in identifying the basal_plate_of_hindbrain and DPallm regions. In the E15.5 sample (Fig. 5D), soFusion significantly outperformed other methods in ARI values, with clustering results highly consistent with manual annotations, particularly for the structures like cartilage, muscle, DPallv, thalamus, subpallium_2, and diencephalon_and_hindbrain. In contrast, Seurat, MultiVI, and spaMultiVAE showed noisier clustering results with fewer meaningful structures identified. Although SpatialGlue and PRESENT effectively separated structures like muscle, thalamus, DPallv, and DPallm, they performed poorly in identifying larger structures such as diencephalon_and_hindbrain and cartilage. In the E18.5 sample (Fig. 5F), soFusion again achieved the highest ARI value, outperforming other methods in identifying subpallium_1, DPallm, diencephalon_and_hindbrain, cartilage_1, and mesenchyme. PRESENT, MultiVI, and spaMultiVAE exhibited fragmented clustering in the diencephalon_and_hindbrain region. SpatialGlue and Seurat, despite getting high ARI values, failed to separate diencephalon_and_hindbrain from mesenchyme. These results demonstrate that soFusion not only accurately identifies brain substructures challenging for single-omics approaches but also achieves highly consistent spatial domain partitioning across different developmental stages. The ARI value for spatial clustering in the E13.5 sample is lower compared to E15.5 and E18.5, which may be due to the finer and more intricate tissue structure in the E13.5 sample, making it more challenging to capture.

Additionally, we observed that in all three samples, the ARI values obtained by soFusion using multi-omics information were significantly higher than those using only ATAC or RNA. ATAC and RNA data exhibited complementary characteristics in identifying specific brain structures. For example, in the E15.5 sample (Fig. 5D), ATAC data excelled in separating DPallv and DPallm, while RNA data provided more precise identification of muscle and cartilage regions. By integrating ATAC and RNA, soFusion successfully achieved accurate identification of these structures. Moreover, soFusion delivered excellent clustering performance in regions difficult to identify with single-omics data, such as subpallium_2 and diencephalon_and_hindbrain. In summary, soFusion excels in integrating spatial multi-omics information, significantly enhancing the accuracy and consistency of spatial domain identification through multimodal data complementarity.

### soFusion dissects spatial epigenome–transcriptome for mouse brain samples at higher resolution

Finally, we applied soFusion to the brain coronal dataset from a mouse (postnatal Day 22) generated using spatial ATAC-RNA-seq and CUT&Tag-RNA-seq [10]. This dataset integrates multimodal omics data (ATAC-seq, H3K27me3, H3K4me3, H3K27ac, and transcriptome) and exhibits highly complex neuroanatomical structures, including cortical lamination and fine subregions of basal ganglia.

**Figure 6:**
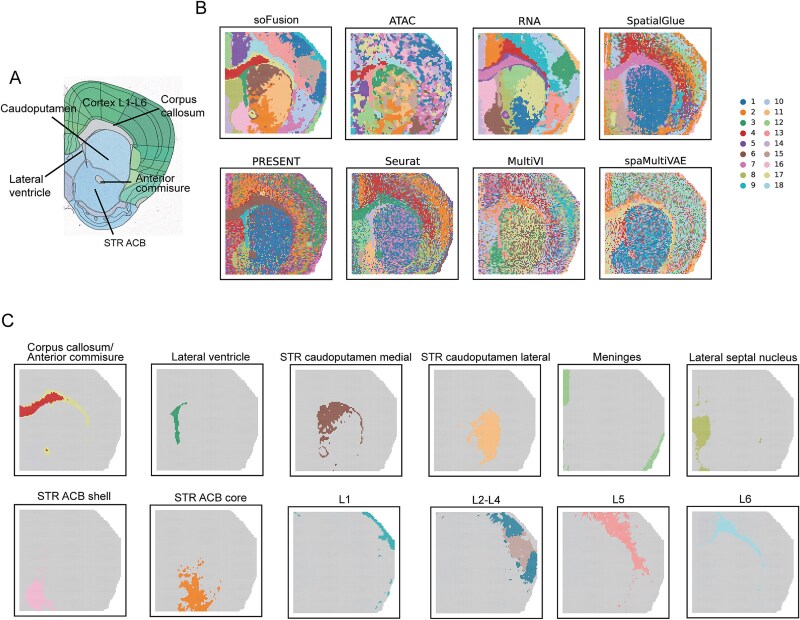
Performance comparisons of different methods on the spatial ATAC-RNA-seq mouse (P22) brain dataset. (A) A mouse brain coronal image from the Allen Mouse Brain Atlas. STR: striatum; ACB: nucleus accumbens. (B) The clustering results of soFusion, SpatialGlue, PRESENT, Seurat, MultiVI, and spaMultiVAE methods, as well as the results of soFusion using only ATAC or RNA. (C) The visualization of some structures of the mouse brain identified by soFusion.

Using the Allen Mouse Brain Atlas [42] as a reference (Fig. 6A), we evaluated the clustering performance of different methods. As shown in Fig. 6B and Supplementary Fig. S3, soFusion-derived spatial domains exhibited minimal noise and closely aligned with anatomical structures. Among five compared methods, SpatialGlue and PRESENT performed relatively well, successfully identifying the lateral ventricle, corpus callosum, and nucleus accumbens (ACB) shell, while capturing cortical layered structures (Fig. 6B). However, these methods failed to distinguish adjacent regions such as the caudoputamen and ACB core. In contrast, soFusion not only identified these regions but further subdivided the caudoputamen into medial and lateral subregions. The medial caudoputamen lies closer to the midline, whereas the lateral subregion is positioned near the cortex. These subregions show differences in motor regulation and learning tasks [43], demonstrating soFusion’s capability to resolve anatomically similar structures (Fig. 6B).

Based on previous studies [42], we annotated soFusion-identified regions (Fig. 6C). The annotations revealed high consistency with the Allen Mouse Brain Atlas for structures like the corpus callosum and lateral ventricle. Distinct laminar structures (L1-L6) were also observed. Additional tests combining RNA data with three histone modification datasets confirmed soFusion’s precision in resolving cortical layers. These results demonstrate that soFusion effectively resolves spatial associations in multi-modal omics data and distinguishes adjacent brain regions (e.g. caudoputamen subregions versus ACB core). Its stability and accuracy in high-resolution spatial domain identification validate the effectiveness of this method for integrating cross-omics data and revealing spatial heterogeneity in complex biological systems.

## Discussion

Spatial multi-omics technologies enable the acquisition of multi-dimensional molecular features from a tissue section, offering new perspectives for understanding cellular functions and tissue structures [44–46]. Despite their great potential in biological research, these technologies face significant challenges in data integration and analysis. The data are derived from different omics layers, which not only differ fundamentally in type but also exhibit distinct spatial distribution patterns. Existing methods typically focus on single-omics data analysis or provide coarse integration at low dimensions. They fail to effectively explore the synergistic effects between omics and struggle to integrate spatial information in higher dimensions. To address these challenges, we propose soFusion, an efficient spatial multi-omics data integration method, to enhance both the efficiency and accuracy of data integration.

soFusion first embeds features from different omics using GCNs. Given that omics features exhibit both commonalities and specificities, we introduce a novel intra- and inter-omics feature learning strategy. This strategy preserves the relationships between different omics while capturing their individual specificities. To address the issue of differing distribution patterns across omics data, we designed customized decoders for transcriptomics, epigenomics, and proteomics respectively. Additionally, to accommodate other omics data, soFusion integrates a universal decoder based on fully connected networks. We evaluated soFusion on six datasets covering different tissue types and technical platforms. The results show that soFusion effectively integrates various features from different omics and demonstrates high accuracy and strong robustness in spatial domain recognition tasks.

Although soFusion has currently been validated primarily on datasets involving spatial transcriptomics and proteomics, as well as spatial transcriptomics and epigenomics, its modular and flexible design demonstrates the potential for easy extension to integrate three or more omics data types. Moreover, soFusion can also handle spatial multi-omics data comprising multiple tissue slices by adopting a block-diagonal adjacency matrix strategy. Because soFusion uses GCN to obtain low-dimensional embeddings from each omics modality, scaling the model to extremely large datasets are challenging without substantial computational resources. As shown in the Supplementary Fig. S5, both execution time and GPU memory usage scale approximately linearly with the number of spots, reaching ~2.7 min and ~1.3 GB GPU memory for 10 000 spots. In future work, we will explore minibatch training with neighborhood sampling or construct sparse adjacency matrices to improve soFusion’s scalability on larger datasets. With the ongoing advancement of technology and continuous improvements in functionality, soFusion is expected to provide support for uncovering the underlying mechanisms of complex biological systems and make significant contributions to the field of multi-omics data analysis.

**Key Points**

- soFusion is a novel representation learning framework tailored for spatial multi-omics data, enabling precise delineation of tissue architecture.
- soFusion incorporates a novel intra- and inter-omics feature learning strategy that effectively captures both cross-modal relationships and modality-specific features.
- The incorporation of modality-specific decoders within soFusion ensures the preservation of unique information intrinsic to each omics.
- soFusion exhibits enhanced performance in spatial domain identification when applied to different types of data, including gene expression, protein abundance, and epigenetic features.

## Supplementary Material

Supplementary_Materials_bbaf513

## Data Availability

The mouse thymus dataset and human Lymph Node dataset are obtained from https://zenodo.org/records/10362607. The SPOTS mouse spleen dataset is available at GEO with accession code GSE198353. The human tonsil dataset can be accessed at https://doi.org/10.6084/m9.figshare.21623148.v5. The MISAR-seq mouse brain dataset is accessible at the National Genomics Data Center with accession number OEP003285. The spatial ATAC-RNA-seq mouse brain dataset can be found at https://web.atlasxomics.com/visualization/Fan/.

## Code availability

The code of soFusion is available at https://github.com/sunxue-yy/soFusion.
