# Supplementary material for: soFusion: facilitating tissue structure identification via spatial multi-omics data fusion
Source: Brief Bioinform. 2025 Sep 29;26(5):bbaf513. doi: 10.1093/bib/bbaf513 (PMC12477611; doi:10.1093/bib/bbaf513)
Supplement: Supplementary_Materials_bbaf513 [file supplementary_materials_bbaf513.docx]

**Supplementary Materials**

# Platform and Model Settings

## 1.1 Platform

We implement the soFusion on the PyTorch 1.11.0, running on an Ubuntu 20.04 server with an Intel(R) Xeon(R) Gold 6258R CPU and an NVIDIA Quadro GV100 GPU.

## 1.2 Model parameters

Different hyperparameter settings can affect soFusion’s performance. Here, we provide detailed guidance for selecting key hyperparameters. As shown in Supplementary Figure S4A, we set the latent representation dimensionality to 3000, which yielded satisfactory performance on the lymph node dataset. To balance model complexity and performance, we chose 2 GNN layers (Supplementary Figure S4B). For the loss component weights ($a$, $b$, $c$, and $d$), we set them all to 1, as our method is not sensitive to different loss weight combinations (Supplementary Figure S4C). The feature integration parameters $\alpha$, $\beta$, and $\gamma$ were set to 1:1:1 by default, but adjustments may be needed depending on the dataset. We recommend increasing the weight of better-performing modalities and reducing the weight of less informative modalities (Supplementary Figure S4D). For example, for the lymph node dataset, we used $\alpha=5$ for RNA, $\beta=1$ for ADT, and $\gamma=1$ for feature fusion.

For all benchmarking methods, we followed the default hyperparameters and preprocessing methods recommended in the original papers.

# 2. Datasets and Data Processing

## 2.1 Datasets

We validate the performance of our proposed SoFusion using six datasets from different platforms, which can be downloaded from the public website.

1. The mouse thymus dataset is obtained from <https://zenodo.org/records/10362607>. The sample we used in this study contained 4697 spots, 51 proteins and 23,622 genes.
2. The SPOTS mouse spleen dataset is available at GEO with accession code GSE198353. We used two samples from this dataset, containing 2568 and 2768 spots respectively.
3. The human tonsil dataset can be accessed at <https://doi.org/10.6084/m9.figshare.21623148.v5>. This dataset contains 2492 spots, 2000 genes, and 283 proteins.
4. The human Lymph Node dataset is obtained from <https://zenodo.org/records/10362607>. This dataset is collected from a spatial multi-omics study[1] and contains expression levels for 18,085 genes and 31 proteins at 3484 spots.
5. The MISAR-seq mouse brain dataset is accessible at the National Genomics Data Center with accession number OEP003285. We used three samples from this dataset, including E13.5 (1777 spots), E15.5 (1949 spots), and E18.5 (2129 spots).
6. The P22 mouse brain dataset including one spatial ATAC–RNA-seq sample and three spatial CUT&Tag-RNA-seq samples (H3K27me3, H3K4me3 and H3K27ac), and can be found at <https://web.atlasxomics.com/visualization/Fan/>. In four samples, the number of spots ranged from 9,215 to 9,752, the number of genes ranged from 22,731 to 25,881 and the number of peaks ranged from 35,270 to 121,068.

## 2.2 Data processing

For all datasets used in our study, we initially removed spots located outside the main tissue regions. For each modality (RNA, epigenomic, and protein), we first used SCANPY[2] to filter out features expressed in fewer than three spots. Next, the count matrices were normalized and log-transformed using SCANPY. Subsequently, we selected the top 3000 most variable features for RNA and the top 8000 for the epigenome.

# Reference

1. Long Y, Ang KS, Sethi R, et al. Deciphering spatial domains from spatial multi-omics with SpatialGlue. Nat Methods 2024; 21:1658–1667

2. Wolf FA, Angerer P, Theis FJ. SCANPY: large-scale single-cell gene expression data analysis. Genome Biol 2018; 19:15

# Supplementary Tables

**Supplementary Table S1**. Summary of the datasets used in this study.

| Platform | Tissue | Section | #Spots |
| --- | --- | --- | --- |
| Stereo-CITE-seq | Mouse thymus | / | 4697 |
| SPOTS | mouse spleen | Sample 1 | 2568 |
|  |  | Sample 2 | 2768 |
| spatial-CITE-seq | Human tonsil | / | 2492 |
| 10x Visium RNA-Protein | Human Lymph Node | A1 | 3484 |
| MISAR-seq | mouse brain | E13.5 | 1777 |
|  |  | E15.5 | 1949 |
|  |  | E18.5 | 2129 |
| Spatial ATAC-RNA-seq and spatial CUT&Tag-RNA-seq | mouse brain | RNA&ATAC | 9215 |
|  |  | RNA&H3K27me3 | 9752 |
|  |  | RNA&H3K4me3 | 9548 |
|  |  | RNA&H3K27ac | 9370 |

# Supplementary Figures


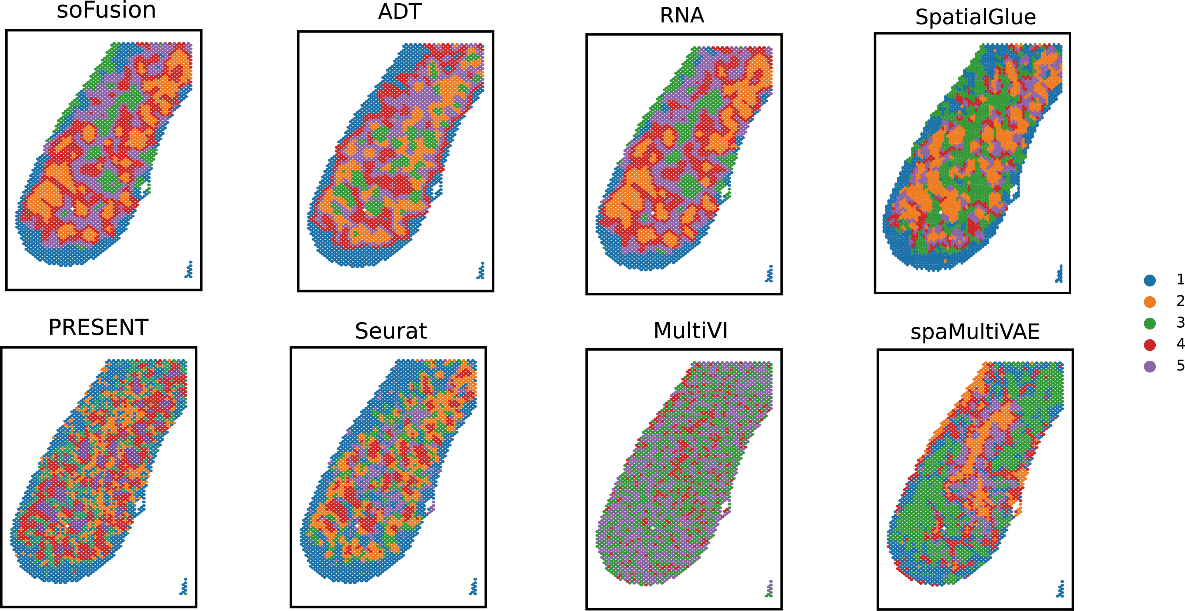


**Figure S1.** The spatial clustering results for the mouse spleen replicate sample by different methods. (Alt Text: Mouse spleen replicate clustering results.)


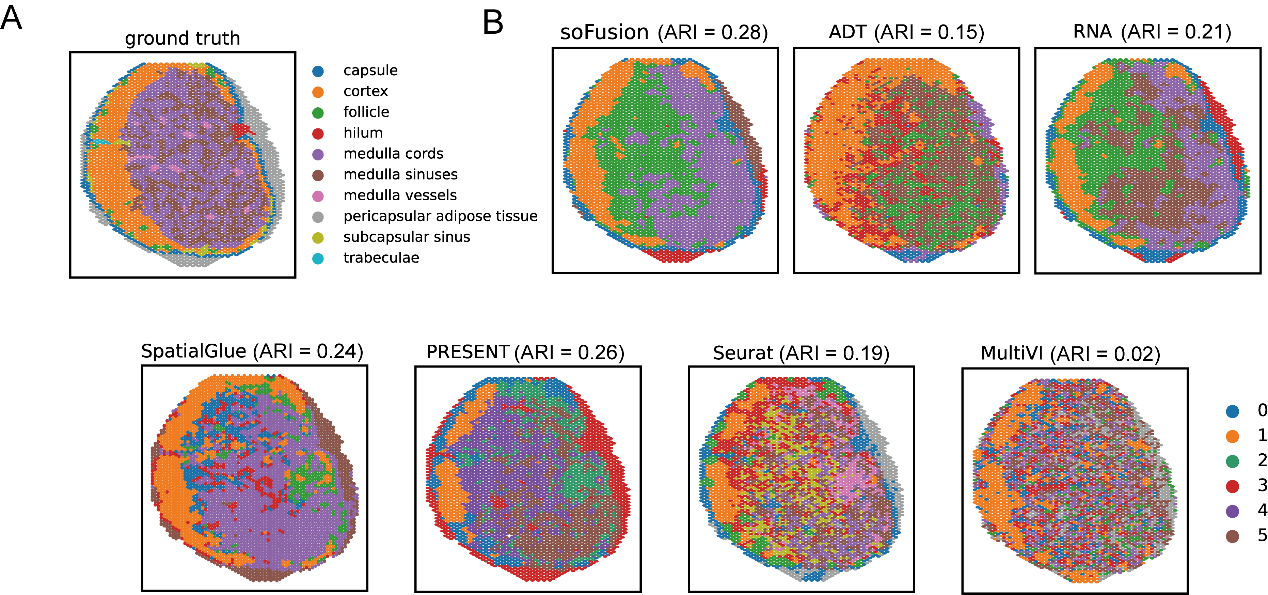


**Figure S2.** The spatial clustering results for lymph node sample A1 by different methods. (Alt Text: Lymph node sample A1 clustering results.)


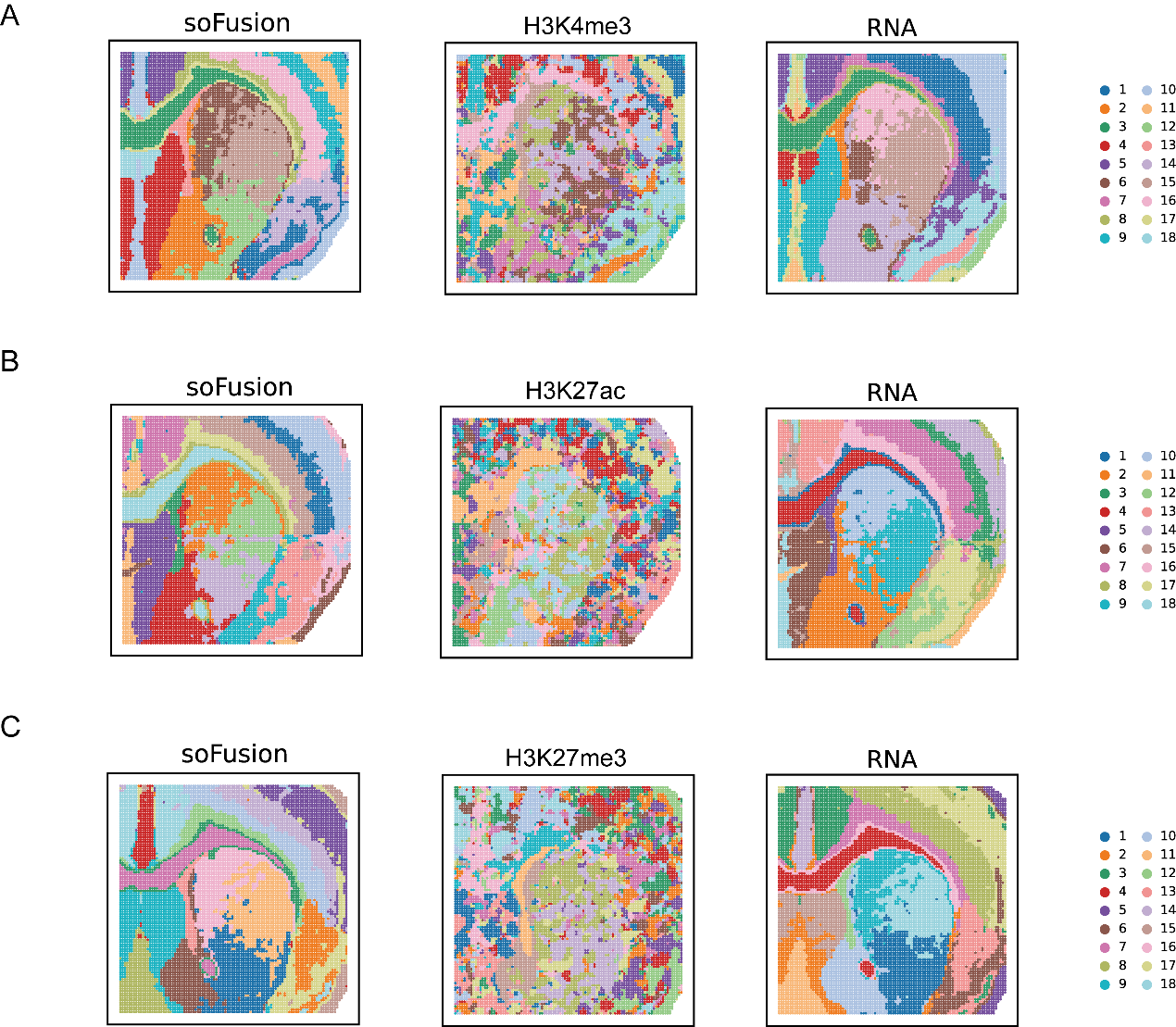


**Figure S3.** The spatial clustering results of SoFusion on mouse brain P22 samples acquired with spatial CUT&Tag-RNA-seq (H3K4me3, H3K27ac and H3K27me3). (Alt Text: Mouse brain P22 samples clustering results.)


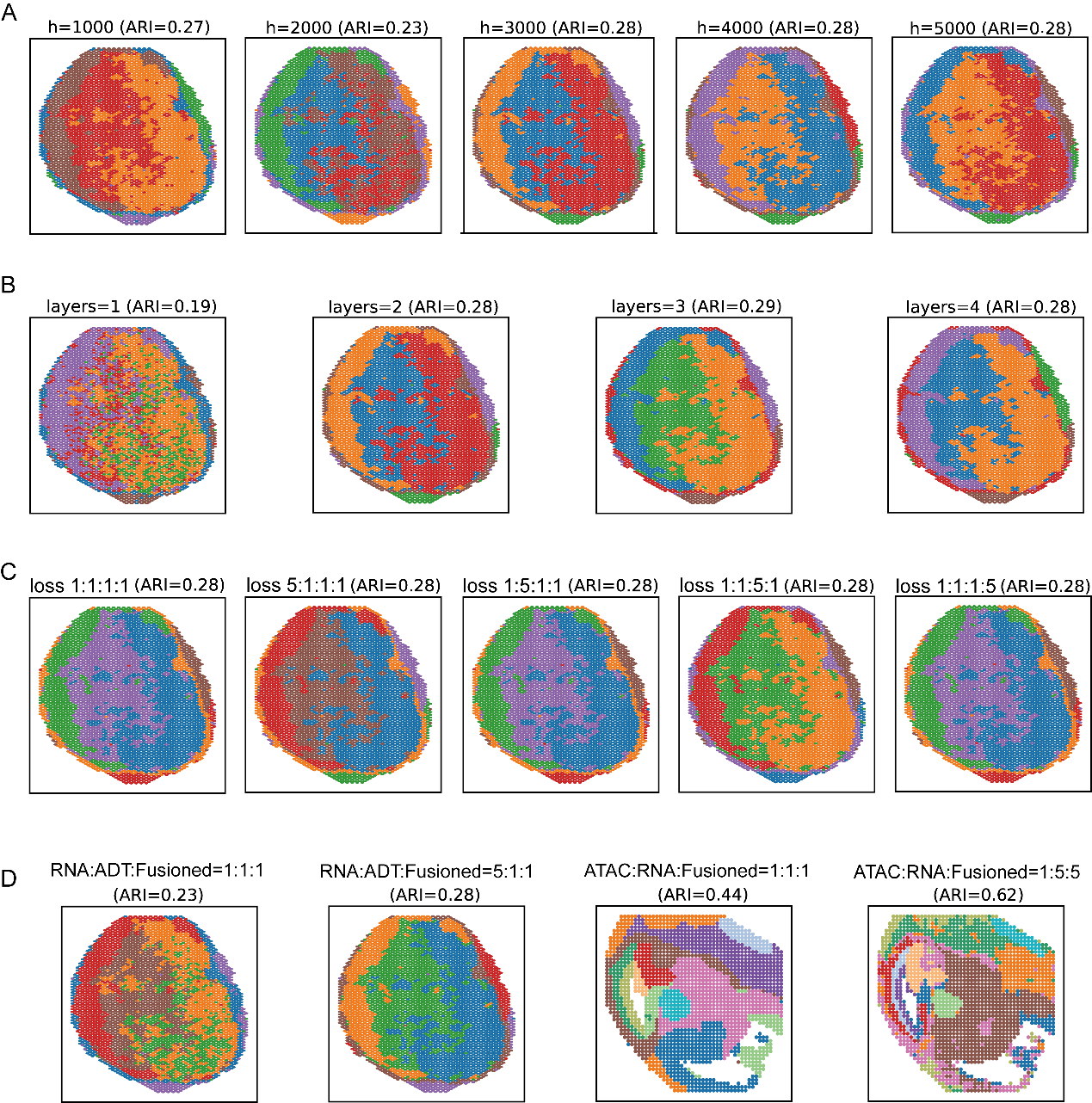


**Figure S4.** Effect of different hyperparameters on soFusion performance. (A) The dimensionality of latent representations. (B) The number of GNN layers. (C) The loss component weights. (D) The feature integration parameters. (Alt Text: Effects of hyperparameters on soFusion performance, including latent dimension, GNN layers, loss weights, and feature integration settings.)


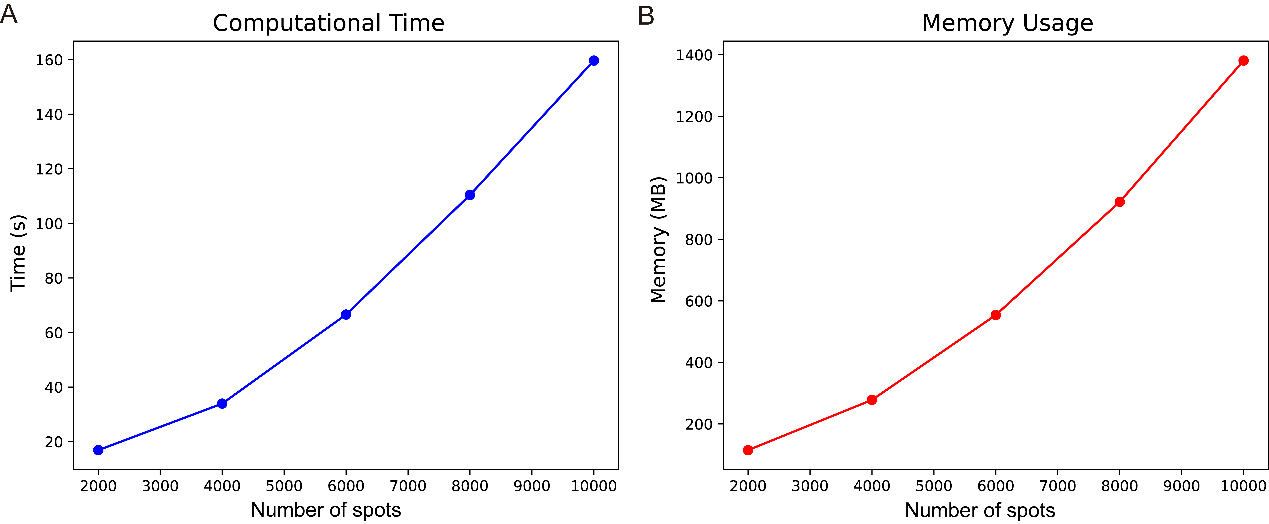


**Figure S5.** Computational cost of soFusion on the simulated dataset. (A) Running time of soFusion. (B) GPU memory usage of soFusion. (Alt Text: soFusion runtime and GPU memory on simulated data.)
